# The Burden of Cerebrovascular Disease in the United States

**DOI:** 10.5888/pcd16.180411

**Published:** 2019-04-25

**Authors:** Xin Tong, Quanhe Yang, Matthew D. Ritchey, Mary G. George, Sandra L. Jackson, Cathleen Gillespie, Robert K. Merritt

**Affiliations:** 1 Division for Heart Disease and Stroke Prevention, National Center for Chronic Disease Prevention and Health Promotion, Centers for Disease Control and Prevention, Atlanta, Georgia

## Abstract

**Introduction:**

Little is known about trends in the overall combined burden of fatal and nonfatal cerebrovascular disease events in the United States. Our objective was to describe the combined burden by age, sex, and region from 2006 through 2014.

**Methods:**

We used data on adults aged 35 and older from 2006 through 2014 Nationwide Emergency Department Sample, National Inpatient Sample of the Healthcare Cost and Utilization Project, and the National Vital Statistics System. We calculated age-standardized cerebrovascular disease event rates by using the 2010 US Census population. Trends in rates were assessed by calculating the relative percentage change (RPC) between 2006 and 2014, and by using Joinpoint to obtain *P* values for overall trends.

**Results:**

The age-standardized rate increased significantly for total cerebrovascular disease events (primary plus comorbid events) from 1,050 per 100,000 in 2006 to 1,147 per 100,000 in 2014 (*P* < .05 for trend). Treat-and-release emergency department visits with comorbid cerebrovascular disease events increased significantly, from 114 per 100,000 in 2006 to 213 per 100,000 in 2014 (RPC of 87%, *P* < .05 for trend). Significant rate increases were identified among adults aged 35 to 64 with an RPC of 19% in primary cerebrovascular disease events, 48% in comorbid cerebrovascular disease events, and 36% in total events.

**Conclusion:**

Our findings have important implications for the increasing cerebrovascular disease burden among adults aged 35 to 64. Focused prevention strategies should be implemented, especially among young adults who may be unaware of existing modifiable risk factors.

**Summary**

**What is already known about this topic?**

Several studies have reported declines in overall cerebrovascular disease mortality, and stalled declines in hospitalizations. Furthermore, there is significant increasing mortality from cerebrovascular disease among young adults aged 35-64 years.

**What is added by this report?**

This study provides the latest burden estimates of fatal and nonfatal cerebrovascular disease events in the United States by age, sex, and region, from 2006 through 2014.

**What are the implications for public health practice?**

The significant increasing burden in cerebrovascular disease events among persons aged 35 to 64, suggests urgency in extending prevention efforts to a younger population.

## Introduction

Cerebrovascular disease often manifests in a person experiencing an acute nonfatal event (ie, emergency department [ED] visit, hospitalization) or fatal event, with stroke being the primary disease type (1,2). Cerebrovascular disease is a leading cause of serious long-term disability, and the second leading cause of death worldwide (3). Despite declines in cerebrovascular disease mortality rates in the United States since the early 1900s, stroke is the fourth leading cause of death among women and the fifth leading cause of death among men (4). Approximately 795,000 new or recurrent acute strokes occur every year, with an estimated annual direct medical cost for stroke of $17.9 billion in 2012 to 2013 (4). Studies have suggested that previous declines in cerebrovascular disease mortality and hospitalization rates have stalled, and that mortality from cerebrovascular disease is increasing significantly among younger adults (35–64 y) (5,6).

Despite the large public health burden of cerebrovascular disease, no surveillance system exists to collectively track fatal and nonfatal events attributed to cerebrovascular disease or describe the potential shift in burden of these events among different demographic groups in the United States. The aim of our study was to help address this limitation in public health surveillance by estimating the US burden of fatal and nonfatal cerebrovascular disease events (a combination of the mutually exclusive estimates of treat-and-release emergency department [ED] visits, nonelective acute nonfatal hospitalizations, and deaths) by age, sex, and region, from 2006 through 2014. The findings and methodology presented in this study can be used to guide efforts to decrease the health care and mortality burden of cerebrovascular disease in the United States and to track the progress of these efforts.

## Methods

### Data sources and study sample

We used the Nationwide Emergency Department Sample (NEDS) (7) and the National (Nationwide) Inpatient Sample (NIS) (8) to examine ED visits and hospitalizations among adults aged 35 or older from 2006 through 2014. NEDS and NIS are part of the Healthcare Cost and Utilization Project sponsored by the Agency for Healthcare Research and Quality (AHRQ). NEDS is the largest all-payer ED database in the United States, yielding national estimates of hospital-based ED visits (7). NIS is a database of hospital inpatient stays derived from billing data submitted by hospitals to statewide data organizations across the United States (8).

We used the *International Classification of Diseases, Ninth Revision, Clinical Modification* (ICD-9-CM) (9) or Clinical Classification Software (CCS) codes created by AHRQ to identify the conditions for ED visits and hospitalizations. Cerebrovascular disease was identified by using CCS codes 109, 110, 111, and 113 for NEDS and NIS.

We used data from the National Vital Statistics System from 2006 to 2014 to examine mortality that derived from death certificates filed in every US state and the District of Columbia (10). The *International Classification of Diseases Tenth Revision* (ICD-10 codes I60–I69) (11) were used to identify either the underlying cause or the contributing cause of death as cerebrovascular disease.

Our analyses did not include transient ischemic attack (TIA) events.

### Statistical methods

For NEDS and NIS, the unit of analysis was the hospital discharge; we estimated the number of cerebrovascular disease events that occurred from 2006 through 2014. To account for the redesign of NIS in 2012, we used trend weights developed by AHRQ to make estimates comparable for data prior to 2012 (12).

Primary cerebrovascular disease events (where cerebrovascular disease was listed as the primary cause of the ED encounter or hospitalization or as the underlying cause of death) and comorbid cerebrovascular disease events (where cerebrovascular disease was listed either as a comorbid condition or as a contributing cause of death) were examined independently. We used methods developed by the Million Hearts initiative to estimate the total number of fatal and nonfatal events that occurred nationally (13). Applying these methods, we defined the mutually exclusive cerebrovascular disease events as the sum of the events calculated independently for ED visits, hospitalizations, and deaths after applying criteria to exclude double counting. We excluded ED visits where the patient died in the ED, was transferred to another hospital, or was admitted to the same hospital. We excluded hospitalizations if they were designated as elective, if the patient died in the hospital, or if the patient was transferred to another hospital. We calculated acute event rates by using intercensal population estimates as the denominators, standardized to the 2010 US population (13–15). We also derived estimates of total cerebrovascular disease events by age (35–64 y, 65–74 y, 75–84 y, and ≥85 years), sex, and region (Northeast, Midwest, South, and West). We calculated the relative percentage changes (RPCs) along with 95% confidence intervals (CIs) for event rates between 2006 and 2014, and we used Joinpoint software (version 4.3.1.0, National Cancer Institute) to conduct trend analyses based on age-standardized mutually exclusive rates from 2006 through 2014. Joinpoint regression fits a series of joined straight lines on a logarithmic scale to the trend data, and *P* < .05 was considered significant.

We used SAS 9.3-callable SUDAAN (RTI International) to account for the multistage, disproportionate stratified sampling design for NEDS and NIS. Because NEDS, NIS, and National Vital Statistics System data are publicly available and do not contain direct personal identifiers, this study was exempt from review by the institutional review board of the Centers for Disease Control and Prevention.

## Results

From 2006 through 2014, the age-standardized mutually exclusive rates for events with cerebrovascular disease as the primary cause remained stable (RPC, −2%) (Table 1). However, the rates for comorbid cerebrovascular disease events increased significantly (RPC, 16%; *P* < .05 for trend) (Table 2), and the rates for combined primary and comorbid cerebrovascular disease events increased significantly from 1,050 per 100,000 in 2006 to 1,147 per 100,000 in 2014 (RPC, 9%; *P* < .05 for trend) (Table 3).

**Table 1:** Mutually Exclusive Cerebrovascular Events Where Cerebrovascular Disease Was Listed As the Primary Reason for Emergency Department (ED) Visit or Hospital Admission or As Primary Cause of Death, 2006–2014^a^

Variable	2006	2010	2014	RPC (95% CI)^b^
	n (Rate per 100,000)			
**Total events^c^**	626,897 (409.6)	660,198 (405.5)	711,898 (402.9)	−1.6 (−0.6 to −2.7)
**Treat-and-release ED visits**	15,498 (10.1)	18,462 (11.3)	26,794 (15.3^d^)	51.5 (49.5 to 55.3)
**Nonfatal hospitalizations**	475,308 (309.8)	513,239 (315.2)	552,975 (313.6)	1.2 (−0.2 to 2.7)
**Deaths**	136,090 (89.7)	128,497 (78.9)	132,129 (74.0^d^)	−17.5 (−17.6 to −17.5)
**Age, y**				
35–64	177,713 (150.0)	203,068 (165.7)	220,974 (178.1^d^)	18.7 (15.8 to 22.0)
65–74	124,683 (649.3)	135,489 (624.0)	158,680 (601.1^d^)	−7.4 (−9.6 to −5.1)
75–84	183,761 (1,403.3)	172,202 (1,318.4)	174,343 (1,274.2^d^)	−9.2 (−11.0 to −7.2)
≥85	140,739 (2,892.3)	149,438 (2,720.3)	157,901 (2,562.4^d^)	−11.4 (−12.8 to −9.9)
**Sex^c^**				
Men	285,064 (437.9)	309,385 (437.5)	342,477 (438.4)	0.1 (−0.9 to 1.2)
Women	341,832 (383.4)	350,813 (374.7)	369,421 (368.7^d^)	−3.8 (−4.8 to −2.8)
**Age by sex, y**				
Men 35–64	98,254 (168.9)	113,974 (189.4)	126,301 (207.4^d^)	22.8 (20.0 to 25.9)
Men 65–74	65,293 (737.6)	73,122 (724.2)	85,314 (690.9^d^)	−6.3 (−8.6 to −3.9)
Men 75–84	79,319 (1,479.1)	76,373 (1,394.5)	80,231 (1,361.4^d^)	−8.0 (−5.8 to −9.9)
Men ≥85	42,199 (2779.2)	45,916 (2565.6)	50,631 (2,400.8^d^)	−13.6 (−15.0 to −12.1)
Women 35–64	79,459 (131.8)	89,095 (142.8)	94,673 (149.9^d^)	13.7 (10.7 to 17.2)
Women 65–74	59,391 (573.8)	62,367 (536.9)	73,366 (522.2^d^)	−9.0 (−11.0 to −6.9)
Women 75–84	104,443 (1,350.7)	95,829 (1,263.5)	94,111 (1,208.2^d^)	−10.6 (−12.2 to −8.8)
Women ≥85	98,540 (2,943.6)	103,522 (2,795.1)	107,270 (2,646.5^d^)	−10.1 (−11.4 to −8.7)
**Region^c^**				
Northeast	114,991 (372.0)	119,121 (373.1)	12,7371 (379.5)	2.0 (−1.2 to 5.6)
Midwest	144,831 (410.6)	145,724 (396.7)	159,581 (411.7)	0.3 (−1.2 to 1.8)
South	248,350 (454.0)	265,823 (450.2)	289,043 (443.3)	−2.4 (−4.2 to −0.4)
West	118,902 (369.7)	129,681 (369.3)	135,948 (346.8)	−6.2 (−7.5 to −4.8)

Abbreviations: CI, confidence interval; RPC, relative percentage change.

a Sources: Nationwide Emergency Department Sample (7), National Inpatient Sample (8), and National Vital Statistics System (10).

b RPC was calculated by dividing 2014–2006 rates by 2006 rates and is based on the rate per 100,000 persons.

c Standardized by age to the 2010 US Census population distribution among adults aged ≥35 years. The crude age–sex specific rates were reported for subgroup estimate.

d *P* < .05 for trend from 2006 through 2014 based on Joinpoint analyses.

**Table 2:** Mutually Exclusive Comorbid Events Where Cerebrovascular Disease Was Listed As a Secondary Condition or Contributing Cause of Death, 2006–2014^a^

Variable	2006	2010	2014	RPC (95% CI)^b^
	n (Rate per 100,000)			
**Total^c^**	979,953 (639.8)	1,196,840 (735.0)	1,317,223 (743.7^d^)	16.2 (15.7 to 16.8)
**Treat-and-release ED visits**	174,394 (113.5)	264,592 (162.5)	374,375 (212.5^d^)	87.1 (84.7 to 89.4)
**Nonfatal hospitalizations**	712,109 (464.8)	845,172 (519.1)	852,830 (480.8)	3.5 (1.6 to 5.4)
**Deaths**	93,451 (61.5)	87,076 (53.5)	90,018 (50.4^d^)	−18.1 (−18.1 to −18.1)
**Age, y**				
35–64	237,907 (200.8)	321,202 (262.1)	368,709 (297.2^d^)	48.0 (46.5 to 49.7)
65–74	215,222 (1,120.8)	268,193 (1,235.1)	317,818 (1,203.9)	7.4 (6.1 to 8.9)
75–84	322,629 (2,463.7)	355,011 (2,718.1)	364,045 (2,660.6)	8.0 (7.0 to 9.1)
≥85	204,196 (4,196.4)	252,435 (4,595.2)	266,651 (4,327.2)	3.1 (2.4 to 3.9)
**Sex^c^**				
Men	446,213 (693.6)	552,992 (791.5)	624,041 (802.9)	15.8 (15.1 to 16.5)
Women	533,740 (600.0)	643,848 (691.6)	693,182 (696.6)	16.1 (15.7 to 16.5)
**Age by sex**				
Men 35–64	125,214 (215.2)	167,036 (277.6)	193,513 (317.7^d^)	47.7 (45.9 to 49.6)
Men 65–74	109,558 (1,237.6)	138,226 (1,369.0)	162,750 (1,317.9)	6.5 (4.8 to 8.3)
Men 75–84	142,152 (2,650.9)	159,260 (2,907.9)	169,198 (2,871.0)	8.3 (7.0 to 9.7)
Men ≥85	69,289 (4,563.3)	88,471 (4,943.4)	98,580 (4,674.5)	2.4 (1.5 to 3.5)
Women 35–64	112,693 (187.0)	154,166 (247.1)	175,196 (277.5^d^)	48.4 (47.0 to 49.9)
Women 65–74	105,664 (1,020.8)	129,967 (1,118.8)	155,069 (1,103.7)	8.1 (7.1 to 9.2)
Women 75–84	180,476 (2,333.9)	195,750 (2,581.0)	194,847 (2,501.5)	7.2 (6.3 to 8.1)
Women ≥85	134,907 (4,030.0)	163,964 (4,427.0)	168,071 (4,146.5)	2.9 (2.3 to 3.6)
**Region^c^**				
Northeast	199,037 (643.5)	204,071 (638.9)	220,620 (656.6)	2.0 (1.6 to 2.5)
Midwest	242,528 (686.8)	293,623 (799.4)	334,282 (862.8^d^)	25.6 (25.3 to 26.0)
South	368,695 (673.0)	474,752 (802.5)	503,251 (768.0^d^)	14.1 (12.1 to 16.4)
West	169,728 (529.0)	224,467 (641.4)	259,186 (660.7^d^)	24.9 (24.1 to 25.6)

Abbreviations: CI, confidence interval; ED, emergency department; RPC, relative percentage change.

a Sources: Nationwide Emergency Department Sample (7), National Inpatient Sample (8), and National Vital Statistics System (10).

b RPC was calculated by dividing 2014–2006 rates by 2006 rates and is based on the rate per 100,000 persons.

c Standardized by age to the 2010 US Census population distribution among adults aged ≥35 years. The crude age–sex specific rates were reported for subgroup estimate.

d *P* <.05 for trend from 2006 through 2014 based on Joinpoint analyses.

**Table 3:** Mutually Exclusive Total Cerebrovascular Disease Events (Primary and Comorbid), by Age, Sex, and Region, 2006–2014^a^

Variable	2006	2010	2014	RPC (95% CI)^b^
	n (Rate per 100,000)			
**Total^c^**	1,606,850 (1,049.5)	1,857,038 (1,140.5)	2,029,122 (1,146.6^d^)	9.3 (8.5 to 10.1)
**Treat-and-release ED visits**	189,892 (123.6)	283,054 (173.8)	401,169 (227.8^d^)	84.3 (82.0 to 86.3)
**Nonfatal hospitalizations**	1,187,417 (774.6)	1,358,411 (834.3)	1,405,806 (7,94.4)	2.6 (1.0 to 4.2)
**Deaths**	229,541 (151.3)	215,573 (132.4)	222,147 (124.4^d^)	−17.7 (−17.7 to −17.7)
**Age, y**				
35–64	415,620 (350.9)	524,270 (427.8)	589,683 (475.4^d^)	35.5 (33.4 to 37.8)
65–74	339,905 (1,770.1)	403,682 (1,859.1)	476,499 (1,805.0)	2.0 (0.3 to 3.8)
75–84	506,390 (3,867.0)	527,213 (4,036.5)	538,388 (3,934.8)	1.8 (0.3 to 3.3)
≥85	344,934 (7,088.8)	401,873 (7,315.5)	424,552 (6,889.6)	−2.8 (−3.9 to −1.6)
**Sex^c^**				
Men	731,277 (1,131.5)	862,377 (1,229.1)	966,518 (1,241.3^d^)	9.7 (8.8 to 10.6)
Women	875,572 (983.5)	994,661 (1,066.3)	1,062,603 (1,065.3^d^)	8.3 (7.6 to 9.0)
**Age by sex, y**				
Men 35–64	223,468 (384.0)	281,009 (467.0)	319,814 (525.1^d^)	36.7 (34.5 to 39.1)
Men 65–74	174,851 (1,975.2)	211,348 (2,093.3)	248,064 (2,008.8)	1.7 (−0.3 to 3.9)
Men 75–84	221,471 (4,130.0)	235,633 (4,302.4)	249,430 (4,232.4)	2.5 (0.9 to 4.2)
Men ≥85	111,488 (7,342.5)	134,387 (7,509.0)	149,210 (7,075.4)	−3.6 (−4.9 to −2.2)
Women 35–64	192,152 (318.8)	243,261 (390.0)	269,869 (427.4^d^)	34.1 (32.0 to 36.3)
Women 65–74	165,054 (1,594.6)	192,334 (1,655.6)	228,435 (1,626.0)	2.0 (0.5 to 3.5)
Women 75–84	284,919 (3,684.6)	291,579 (3,844.5)	288,958 (3,709.7)	0.7 (−0.6 to 2.1)
Women ≥85	233,447 (6,973.7)	267,487 (7,222.0)	275,342 (6,792.9)	−2.6 (−3.6 to −1.5)
**Region^c^**				
Northeast	287,964 (930.4)	292,646 (920.8)	307,323 (912.8)	−1.9 (−5.0 to 1.6)
Midwest	339,388 (961.1)	368,514 (1,011.7)	382,947 (985.5)	2.5 (0.5 to 4.7)
South	546,017 (998.6)	617,905 (1,063.5)	656,794 (1,003.8)	0.5 (−1.8 to 3.1)
West	259,291 (808.7)	287,945 (837.6)	307,818 (784.8)	−3.0 (−4.7 to −1.1)

Abbreviations: CI, confidence interval; ED, emergency department; RPC, relative percentage change.

a Sources: Nationwide Emergency Department Sample (7), National Inpatient Sample (8), and National Vital Statistics System (10).

b RPC was calculated by dividing 2014–2006 rates by 2006 rates and is based on the rate per 100,000 persons.

c Standardized by age to the 2010 US Census population distribution among adults aged ≥35 years. The crude age–sex specific rates were reported for subgroup estimate.

d *P* < .05 for trend from 2006 through 2014 based on Joinpoint analyses.

Approximately 0.7 million mutually exclusive events occurred with cerebrovascular disease as the primary cause in 2014. The treat-and-release ED visit rates increased significantly, from 10 per 100,000 in 2006 to 15 per 100,000 in 2014 (RPC, 52%; *P* < .05 for trend), whereas the age-standardized rate of acute nonfatal hospitalizations did not change (RPC, 1%) during the same time period (Table 1). The age-standardized rate of underlying cause of death attributable to cerebrovascular disease declined significantly, from 90 per 100,000 in 2006 to 74 per 100,000 in 2014 (RPC, −18%; *P* < .05 for trend). The mutually exclusive primary cerebrovascular disease event rate declined significantly among people aged 65 to 74 (RPC, −7%), aged 75–84 (RPC, −9%), and aged 85 or older (RPC, −11%; *P* < .05 for all trends) (Table 1) from 2006 through 2014. However, a substantial increase occurred among those aged 35 to 64, with the rate increasing from 150 per 100,000 to 178 per 100,000 (RPC, 19%; *P* < .05 for trend). There was no change over time among men, but there was a significant decrease in age-standardized rates among women (RPC, −4%; *P* < .05 for trend). When examining the event rates by age and sex, rates for men aged 35 to 64 increased the most (RPC, 23%; *P* < .05 for trend), followed by women aged 35 to 64 (RPC, 14%, *P* < .05 for trend). There were no differences in event rates by census region (Table 1).

Approximately 1.3 million mutually exclusive events with cerebrovascular disease listed as a comorbid condition occurred in 2014 (Table 2). The mutually exclusive treat-and-release ED visit rates with cerebrovascular disease as a comorbid condition increased significantly from 2006 (114 per 100,000) through 2014 (213 per 100,000) (RPC, 87%; *P* < .05 for trend). No change occurred in the age-standardized rate of acute nonfatal hospitalizations across the study period (RPC, 4%). The age-standardized rate of cerebrovascular disease listed as the contributing cause of death declined significantly, from 62 per 100,000 to 50 per 100,000 (RPC, −18%; *P* < .05 for trend). During this time, comorbid cerebrovascular disease event rates increased significantly, from 201 per 100,000 to 297 per 100,000 (RPC, 48%; *P* < .05 for trend) among those aged 35 to 64, whereas the event rates were stable among those aged 65 to 74, 75 to 84, and 85 or older. The Midwest, South, and West census regions also had significant increases in comorbid cerebrovascular disease event rates (RPC, 26%, 14%, and 25% respectively; *P *< .05 for all trends) from 2006 through 2014.

Hospitalizations were the most common events that occurred when assessed by event type (primary vs comorbid events), age group, and year (Figure). The percentage of deaths increased with age and was greater for primary cerebrovascular disease events than for comorbid disease events. The percentage of events that were treat-and-release ED encounters increased over time across all age groups for both primary and comorbid disease events, but remained highest for comorbid disease events.

**Figure:**
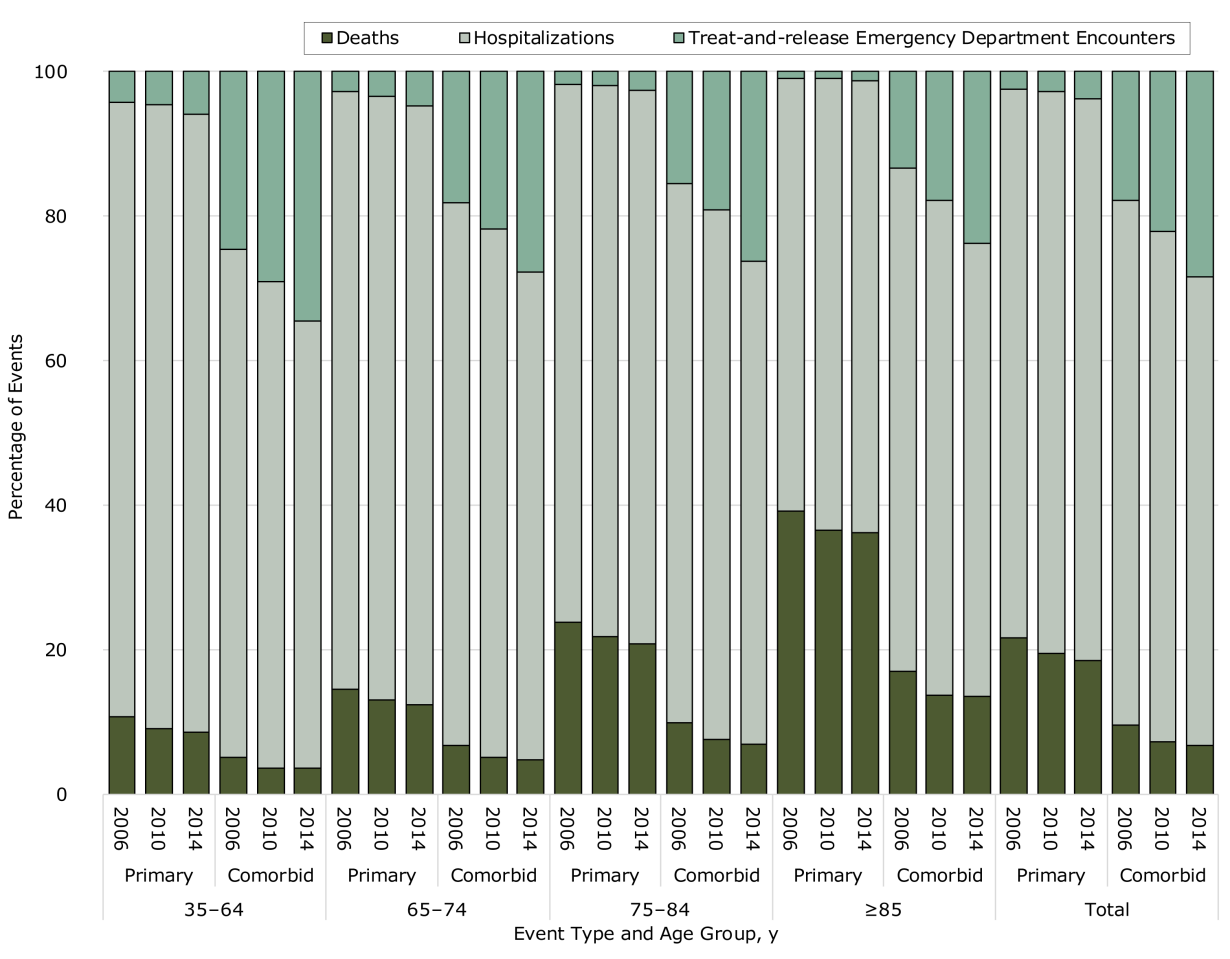
Percentage of cerebrovascular disease events that were treat-and-release ED encounters, hospitalizations, and deaths, by event type, age group, and year, Nationwide Emergency Department Sample, National Inpatient Sample, and National Vital Statistics System, 2006–2014. Event types include primary cerebrovascular events, where cerebrovascular disease was listed as the primary cause of the ED encounter or hospitalization or as the underlying cause of death, and comorbid cerebrovascular events, where cerebrovascular disease was listed either as a comorbid condition or as a contributing cause of death.

**Table:** 

Age, y	Category	Year	Deaths	Hospitalizations	Emergency Department Visits
35–64	Primary	2006	11	85	4
		2010	9	86	5
		2014	9	86	6
	Comorbid	2006	5	70	25
		2010	4	67	29
		2014	4	62	35
65–74	Primary	2006	15	83	3
		2010	13	84	3
		2014	12	83	5
	Comorbid	2006	7	75	18
		2010	5	73	22
		2014	5	67	28
75–84	Primary	2006	24	74	2
		2010	22	76	2
		2014	21	77	3
	Comorbid	2006	10	75	15
		2010	8	73	19
		2014	7	67	26
≥85	Primary	2006	39	60	1
		2010	37	63	1
		2014	36	63	1
	Comorbid	2006	17	70	13
		2010	14	68	18
		2014	14	63	24
Total	Primary	2006	22	76	2
		2010	19	78	3
		2014	19	78	4
	Comorbid	2006	10	73	18
		2010	7	71	22
		2014	7	65	28

Combined (primary plus comorbid) cerebrovascular disease events totaled more than 2.0 million in 2014. The total event rates increased significantly from 1,050 per 100,000 in 2006 to 1,147 per 100,000 in 2014 (RPC, 9%; *P* < .05 for trend) (Table 3). The total treat-and-release ED visit rates increased 84% from 2006 through 2014 (*P* < .05 for trend), and only 3% for total acute nonfatal hospitalizations. There was a significant decline (RPC, −18%; *P* < .05 for trend) in total death rates from 2006 through 2014. The largest increase in total event rates was among those aged 35 to 64 (RPC, 36%; *P* < .05 for trend), and among men aged 35 to 64 (RPC, 37%; *P* < .05 for trend) and women aged 35 to 64 (RPC, 34%; *P* < .05 for trend) from 2006 to 2014.

## Discussion

Our data provide the latest estimates on the total fatal and nonfatal cerebrovascular disease events from 2006 through 2014 in the United States. In 2014, a combined total of 2.0 million cerebrovascular disease events occurred, including approximately 0.7 million mutually exclusive primary cerebrovascular disease events and 1.3 million mutually exclusive comorbid cerebrovascular disease events. Overall, no change occurred in the burden of primary cerebrovascular disease event rates, but the rate of the comorbid cerebrovascular disease, and combined burden of primary plus comorbid cerebrovascular events increased.

Notably, we found that among younger adults (35–64 y) the primary cerebrovascular disease event rate increased 19%, the comorbid cerebrovascular disease event rate increased 48%, and the total burden increased 36% from 2006 through 2014. These increases stand in contrast to the findings for older adults (≥65 y), where primary cerebrovascular disease events declined but no change was observed in the comorbid cerebrovascular disease event burden or the combined burden of primary plus comorbid events. The finding of increasing trends in cerebrovascular disease event burden in younger adults is consistent with several studies (6,16–18). The Greater Cincinnati/Northern Kentucky Stroke Study found trends of increasing stroke incidence among those aged 20 to 54, which would carry a potentially greater lifetime burden of disability (16). Towfighi et al identified significant increases in ischemic stroke hospitalizations among adults aged 35 to 44 from 1997 through 2006 (17). George et al reported significant increases in ischemic stroke hospitalizations and associated traditional stroke risk factors for the 2003–2012 period among persons aged 18 to 54 (6). The extensive 27-year population study from the Dijon Stroke Registry showed a significant increase in ischemic stroke incidence in people younger than 55 and a rising prevalence of vascular risk factors, especially among smokers (18). Recently, a CDC study reported a deceleration in the decline of stroke death rates from 2013 through 2015 with nearly one-third of the estimated excess stroke deaths (representing the hypothetical achievable stroke death reduction) attributable to the slowdown occurring among adults aged 35 to 64 (5). The overall deceleration of stroke mortality in the CDC study is consistent with our finding of no change in the mutually exclusive primary cerebrovascular disease event rates for ED visits, hospitalizations, and deaths.

The changes in the increasing trends in both primary and comorbid cerebrovascular disease events in young adults is a concern. A recent publication reported that the prevalence of obesity has more than doubled since the late 1980s, and more than 1 in 3 US adults (39.8%) were obese in 2015–2016 (19). Obesity is an important risk factor for high blood pressure, which is a primary risk factor for cerebrovascular disease (20). The prevalence of diabetes has more than tripled at the same time. In 2015, 30.3 million (9.4%) US adults had diabetes and 84 million (26%) had prediabetes (21). Diabetes is a significant independent risk factor for cerebrovascular disease (19). In addition, the prevalence of young adults hospitalized with cerebrovascular disease with multiple risk factors has increased significantly in recent years (6). Significant changes in risk profiles in the US population, especially among young adults, as the consequence of obesity and diabetes epidemics, present growing challenges for cerebrovascular disease prevention. These findings should prompt a sense of urgency toward promoting healthy lifestyle behaviors throughout the life course and should encourage awareness, detection, and management of risk factors for cerebrovascular disease.

In our study, treat-and-release ED encounter rates for primary and comorbid cerebrovascular disease events increased significantly from 2006 through 2014 (RPC of 84%). People with a history of cerebrovascular disease are often at increased risk for having subsequent encounters in high-level care settings, including EDs, for multiple reasons (22). These reasons include patient-related factors (eg, development of new health problems, deterioration of overall health status, or complications from stroke), health system factors (eg, poor discharge planning and care coordination), and environmental factors (eg, limited access to care) (23). That the events described in our study were among patients who were treated and released from the ED without being hospitalized indicates that events were likely of lower acuity and potentially avoidable through improved disease management (24). Considerable heterogeneity exists in the research design of studies attempting to determine what interventions are integral to limiting potentially unnecessary use of high-level health care services among adults with cerebrovascular disease (25). However, the interventions with the most robust evidence include use of hospital-initiated support for discharge to home or intermediary care settings (25), use of post-acute and outpatient rehabilitation after having a stroke (23), and use of team-based care models to manage common stroke-related risk factors, such as uncontrolled blood pressure (26).

Substantial disparities exist in age-standardized mortality rates from cerebrovascular disease, with rates highest in the southeastern United States (4,27). In our study, the age-standardized rate for cerebrovascular disease events was the highest in the South census region, but not for comorbid cerebrovascular disease events. The age-standardized mutually exclusive rates of comorbid cerebrovascular disease events increased significantly in the Midwest, South, and West census regions from 2006 through 2014, whereas no differences were observed in primary cerebrovascular disease event rates across all regions. Further studies are needed to explore the reasons for the observed regional differences in cerebrovascular disease event rates, including the disparities in access to care among survivors of cerebrovascular disease.

The strength of this study is that it provides national estimates for the United States; however, our findings have limitations. First, NEDS and NIS are event-level databases; therefore, patient-level data could not be identified, and we were not be able to identify repeat ED visits or hospitalizations in any calendar year. Second, the burden of encounters relating to cerebrovascular disease in outpatient clinical settings could not be evaluated, thereby underestimating the overall burden of cerebrovascular disease on the entire health care system. Third, changes in coding practices might affect temporal trends in mutually exclusive event rates. The increased use of magnetic resonance imaging and enhanced computer tomography of the brain may lead to an increase in diagnosed cerebrovascular disease. Lastly, we excluded ED visits with a primary reason as “acute stroke and discharged to home” in our mutually exclusive cerebrovascular disease event calculation. Patients with acute cerebrovascular disease would likely be admitted to a hospital.

Our findings demonstrate a significantly increased burden in cerebrovascular disease events among persons aged 35 to 64 over the period analyzed. Cerebrovascular disease can substantially be prevented through public health and clinical strategies to promote awareness of risk factors, healthy lifestyle behaviors, risk factor prevention strategies through environmental efforts and policy changes, aggressive management of risk factors, early diagnosis of disease, and personal adoption of healthy lifestyle behaviors across the lifespan. Our findings indicate that to address the increased burden of cerebrovascular disease, prevention efforts need to be expanded to a younger population, specifically those aged 35 to 64.
